# Seeking and Providing Social Support on Twitter for Trauma and Distress During the COVID-19 Pandemic: Content and Sentiment Analysis

**DOI:** 10.2196/46343

**Published:** 2023-08-31

**Authors:** Yildiz Esener, Terika McCall, Adnan Lakdawala, Heejun Kim

**Affiliations:** 1 Department of Information Science University of North Texas Denton, TX United States; 2 Division of Health Informatics Department of Biostatistics Yale School of Public Health New Haven, CT United States; 3 Section of Biomedical Informatics and Data Science Yale School of Medicine New Haven, CT United States; 4 Center for Interdisciplinary Research on AIDS Yale School of Public Health New Haven, CT United States; 5 Elimu Informatics El Cerrito, CA United States

**Keywords:** COVID-19, social support, trauma, distress, posttraumatic stress disorder, PTSD, Twitter, social media, mental health

## Abstract

**Background:**

The COVID-19 pandemic can be recognized as a traumatic event that led to stressors, resulting in trauma or distress among the general population. Social support is vital in the management of these stressors, especially during a traumatic event, such as the COVID-19 pandemic. Because of the limited face-to-face interactions enforced by physical distancing regulations during the pandemic, people sought solace on social media platforms to connect with, and receive support from, one another. Hence, it is crucial to investigate the ways in which people seek and offer support on social media for mental health management.

**Objective:**

The research aimed to examine the types of social support (eg, emotional, informational, instrumental, and appraisal) sought and provided for trauma or distress on Twitter during the COVID-19 pandemic. In addition, this study aimed to gain insight into the difficulties and concerns of people during the pandemic by identifying the associations between terms representing the topics of interest related to trauma or distress and their corresponding sentiments.

**Methods:**

The study methods included content analysis to investigate the type of social support people sought for trauma or distress during the pandemic. Sentiment analysis was also performed to track the negative and positive sentiment tweets posted between January 1, 2020, and March 15, 2021. Association rule mining was used to uncover associations between terms and sentiments in tweets. In addition, the research used Kruskal-Wallis and Mann-Whitney *U* tests to determine whether the retweet count and like count varied based on the social support type.

**Results:**

Most Twitter users who indicated trauma or distress sought emotional support. Regarding sentiment, Twitter users mostly posted negative sentiment tweets, particularly in January 2021. An intriguing observation was that wearing masks could trigger and exacerbate trauma or distress. The results revealed that people mostly sought and provided emotional support on Twitter regarding difficulties with wearing masks, mental health status, financial hardships, and treatment methods for trauma or distress. In addition, tweets regarding emotional support received the most endorsements from other users, highlighting the critical role of social support in fostering a sense of community and reducing the feelings of isolation during the pandemic.

**Conclusions:**

This study demonstrates the potential of social media as a platform to exchange social support during challenging times and to identify the specific concerns (eg, wearing masks and exacerbated symptoms) of individuals with self-reported trauma or distress. The findings provide insights into the types of support that were most beneficial for those struggling with trauma or distress during the pandemic and may inform policy makers and health organizations regarding better practices for pandemic response and special considerations for groups with a history of trauma or distress.

## Introduction

### Background

The COVID-19 pandemic has had a significant global impact, resulting in a substantial loss of life, with a death toll approaching 7 million [1]. In addition to the immediate physical consequences, this crisis had profound implications for mental well-being and gave rise to widespread trauma or distress among individuals at a global level [2,3]. Distress encompasses a wide spectrum of emotional and psychological reactions, including fear, anxiety, sadness, and a sense of being overwhelmed. Comparatively, trauma involves experiences of actual or perceived harm that elicit intense fear, helplessness, or horror [4]. Particularly in times of crisis, such as the COVID-19 pandemic, trauma and distress frequently emerge as prevalent emotional responses, potentially predisposing individuals to the development of posttraumatic stress disorder (PTSD) [3].

Exposure to traumatic events has consistently been associated with a significant increase in the risk of developing PTSD, as evidenced by epidemiological data indicating that 8% to 18% of the individuals who experience trauma subsequently develop this debilitating condition [5]. Previous instances of pandemics have similarly revealed the emergence of PTSD symptoms among affected individuals; for example, approximately 10% of the medical staff who worked during the 2003 severe acute respiratory syndrome (SARS) outbreak in Hong Kong, China, reported having symptoms of PTSD [6]. The 2009 influenza A (H1N1) pandemic resulted in approximately 2% of Chinese university students exhibiting symptoms of PTSD [7]. A study of the long-term psychiatric morbidities associated with SARS survivors revealed PTSD to be the most common condition, and 47.8% of those being treated for SARS 30 months after the SARS outbreak were experiencing PTSD [8].

There is also evidence that the COVID-19 pandemic can be recognized as a traumatic event that led to the general population experiencing trauma, distress, or PTSD [9]. An upsurge in symptoms of psychological distress, including anxiety, depression, self-harm, suicide attempts [10], and PTSD [11], was observed in preliminary reports from China related to the COVID-19 outbreak and the subsequent implementation of quarantine measures. Similarly, during the initial phase of the pandemic, in Italy, a positive correlation was found between the duration of the lockdown and heightened levels of psychological distress, including an increased incidence of PTSD symptoms [12]. In addition, a recent meta-analysis focusing on the prevalence rates of psychological distress during the pandemic revealed elevated rates of anxiety (31.9%), depression (33.7%), and stress (29.6%) [13]. According to the results of a longitudinal survey of 3079 frontline doctors during the acceleration phase of the pandemic, the prevalence rate of psychological distress was 44.7%, whereas the prevalence rate of trauma reached 23.7%. The prevalence rate of probable PTSD was 12.6% at the peak of the pandemic [14].

Social support plays a crucial role in helping individuals to cope with such stressful life events [15]. Social support is defined as “actual or available social resources in times of need and social groups perceived as being supportive” [16]. It has positive effects on stress levels; acts as a protective factor against health problems; and offers opportunities for gathering information, reducing negative thoughts, and expressing emotions [17]. Social support also provides stability; enhances coping strategies; and fosters positive emotions, social connections, and a sense of connectedness after traumatic experiences [18,19]. Perceived social support, which involves individuals’ beliefs about being supported by others, has been particularly associated with improved adaptation after traumatic experiences and found to be effective in various contexts, such as war, school attacks, and natural disasters [20]. According to a study by Hyman et al [21], social support enhances resilience by providing guidance and helping individuals to cope with difficulties. Moreover, several studies have indicated that higher levels of social support at the initiation of treatment for PTSD are linked to enhanced treatment response [22-24]. Furthermore, Price et al [25] found that elevated levels of social support provided during the course of treatment was associated with greater reductions in PTSD symptoms.

According to House [26], social support can be classified as emotional, informational, appraisal, or instrumental support. The term *emotional support messages* refer to messages conveying understanding, empathy, care, kindness, trust, or love to a person in distress. The objective of providing *informational support messages* is to aid recipients in coping with significant challenges that are faced within a particular context by providing relevant advice or helpful knowledge. Although *appraisal support messages* also provide information, they differ from *informational support messages* in that the conveyed information also assists recipients in evaluating themselves or their circumstances through comparison. Finally, *instrumental support messages* include direct tangible help, such as assisting with a task or providing financial assistance [26]. Furthermore, providing social support refers to the act of offering assistance, encouragement, comfort, resources, or inclusion in a supportive social network to others, which may improve their well-being and alleviate stress. Seeking social support refers to the act of requesting assistance, encouragement, comfort, resources, or inclusion in a supportive social network [26]. Perceived social support can also reduce the severity of PTSD symptoms during the course of treatment [27]. These findings suggest that social support, particularly when it is present at the outset of treatment and maintained throughout, plays a significant role in improving treatment outcomes for individuals experiencing symptoms of PTSD.

During the pandemic, many people used social media to communicate with, and support, each other [28], especially because of physical distancing requirements [29]. According to Fung et al [30], as individuals sought information and tried to maintain social connections during the pandemic, social media played a crucial role in maintaining these relationships. The pandemic affected how people lived in various ways, one of which was, without a doubt, their use of the internet; for example, among American internet users, social media use increased from 54 minutes daily during the years before the pandemic to 65 minutes daily in 2020 [31]. In addition, the number of tweets related to COVID-19 reached 6,737,875 in January 2020 and dramatically escalated to 110,220,360 in March 2020 [32]. Although excess use of social media can have negative effects on mental health, researchers have reported that using social media to seek support may help to protect against the adverse effects of pandemic stress and the lack of in-person contact [29]. Furthermore, a study examining the relationship between social support and health using Facebook data found that mental and physical health improved when individuals used social media for social support [33]. Specifically, these data indicate that social media can have a positive impact on the well-being of people by facilitating meaningful social connections, especially in times of social isolation and stress [29,34].

### Objectives

Considering that many people use Twitter to communicate with, and support, one another, it is important to explore the ways in which people offer support on social media to individuals experiencing trauma or distress. This research aimed to examine the types of social support (eg, emotional, informational, instrumental, and appraisal) sought and provided for trauma or distress on Twitter during the COVID-19 pandemic. In addition, this study aimed to gain insight into the difficulties and concerns of people during the pandemic by identifying the associations between terms representing the topics of interest related to trauma or distress and their corresponding sentiments.

## Methods

### Data Collection

Given the widespread impact of the COVID-19 pandemic and the hardships it caused, it was anticipated that individuals may manifest symptoms of PTSD without a formal diagnosis. Therefore, we conducted a comprehensive search for publicly available tweets specifically mentioning PTSD symptoms and used them as a proxy measure of trauma and distress. To ensure the inclusion of a diverse range of perspectives and experiences and to avoid bias in data sampling, we implemented a systematic approach to the data collection process.

First, Twitter was selected as the social media platform of choice for this research because it was a communication tool used by people during the COVID-19 pandemic, especially during the lockdown. Banda et al [32] collected a list of Twitter IDs, dating from January 1, 2020, by using the Twitter streaming application programming interface (API) and made it publicly available. The authors used COVID–19-related terms, including “coronavirus,” “2019ncov,” “corona virus,” “COVD19,” “CoronavirusPandemic,” “COVID-19,” “2019nCoV,” “CoronaOutbreak,” and “coronavirus,” to create the list. We collected our data set by using the list of Twitter IDs from Banda et al [32] and the Twitter search API.

Next, using PTSD symptoms as a proxy measure of trauma and distress, tweets posted in English between January 1, 2020, and March 15, 2021, were filtered using the following search strategy within each tweet’s free text: (“diagnose” OR “diagnosed” OR “diagnosis” OR “I have” OR “I had”) AND (“P.T.S.D.” OR “PTSD” OR “post-traumatic stress disorder” OR “posttraumatic stress disorder” OR “post traumatic stress disorder”). On the basis of this systematic search, 2 data sets were collected in this study. The first data set, which consisted of the tweet text, was used for both content and sentiment analyses. The first data set comprised user-related attributes, including user ID; account description; the IDs of followers and friends; and tweet-related attributes, such as tweet ID and tweet text. The second data set was collected to analyze endorsements (ie, retweet count and like count) accumulated over the COVID-19 pandemic’s time frame. The second data set only included the retweet count and like count for corresponding tweets included in the first data set. Therefore, the first data set consisted of tweets posted between January 1, 2020, and March 15, 2021, and the second data set, which consisted of Twitter users’ endorsements of the collected tweets (ie, the first data set), was gathered on August 14, 2022. *Python-twitter*, a Python wrapper for the Twitter API, was used for data collection [35].

### Data Preprocessing

After applying the search strategy, 4747 tweets were collected, and we used a regular expression [36] to search for statements (eg, “I have PTSD” or “I was diagnosed with P.T.S.D.”) to filter the tweets of users self-reporting trauma or distress during the pandemic. According to Coppersmith et al [36], users may use such a statement to seek support from others in their social network, fight the taboo of mental illness, or perhaps explain some of their behavior. Relevant tweets can be obtained using regular expressions (eg, “I was diagnosed with PTSD”) on a large multiyear health-related collection [36]. In other words, we sought users who publicly self-reported that they had experienced PTSD, which was used as a proxy measure of trauma or distress. After using regular expressions, the matching tweets were also manually reviewed by the lead author (YE) to determine whether they indicated a genuine statement of a self-reported mental health condition (eg, trauma or distress). Thus, of the 4747 tweets collected, 2225 (46.87%) remained for analysis after the filtering process. Textbox 1 shows example tweets that indicated that users were experiencing trauma or distress.

**Examples of tweets mentioning posttraumatic stress disorder (PTSD) found via keyword search and regular expression.**

- “I have PTSD directly related to facial trauma. I can only wear a mask for a short period of time, so I do quick trips. People really should not be mocking panic attacks; mental health is not a joke. My mental health is being treated, but I can only do so much while pregnant.”
- “I have PTSD directly related to having my mouth covered in a vicious attack...won’t get into details here. I get a panic attack about half the time I wear a mask...and that’s after ‘practicing’ at home (counselor recommendation) for weeks.”

### Data Analysis

#### Content Analysis

Content analysis was applied to tweets mentioning PTSD. The coding scheme was designed based on the categories of social support (*informational*, *emotional*, *instrumental*, and *appraisal*) developed by House [26]. Moreover, we labeled the tweets to indicate whether they were *seeking* support within those types or whether they were *providing* it.

Content analysis was conducted by 2 members of the research team—TM (health informatics Doctor of Philosophy and mental health disparities researcher) and YE (Master of Education in Educational Psychology and Doctor of Philosophy candidate in Information Science)—who coded the tweets based on the aforementioned coding schemes. As some of the tweets (1011/2225, 45.44%) did not indicate any of the social support types, an additional theme (ie, *general comments and questions*) was created to code those tweets that did not fall into any of the social support categories. In addition, some of the tweets were coded as >1 type of support. The level of agreement between the 2 reviewers for the content analysis was determined using Cohen κ [37]. Intercoder reliability by κ coefficient was 0.87 (95% CI 0.886-0.854; *P*<.001). A κ statistic of 0.81 to 1.00 indicates “almost perfect” strength of agreement [37].

#### Sentiment Analysis

Sentiment analysis is defined as the process of identifying sentiments in text and labeling them as positive, negative, or neutral based on the emotions expressed. Using natural language processing techniques to interpret subjective and unstructured data, sentiment analysis can help one to understand how a particular person or a group of people feel about any topic. In this study, the Flair sentiment classification library [38] was used to explore the sentiment in the tweets. Flair is a cutting-edge natural language processing framework developed to streamline the process of training and disseminating state-of-the-art sequence labeling, text classification, and language models [39]. Flair is built using a recurrent neural network architecture that can help to capture semantic and syntactic information as well as the context of a word from both sides (preceding and following) of the word [39]. As the Flair model was developed to tackle the ambiguous and situation-specific meanings of words as well as to handle informal terms, such as misspellings and abbreviations, we opted to use this model rather than other models. The sentiment classification model was trained using a large data set of informal movie reviews from the Internet Movie Database (IMDb) and exhibited exceptional performance with an accuracy of 89.5% and an *F*_1_-score of 0.89 [38]. The Flair sentiment analysis library returns values ranging from 0 to 1, with the label of positive or negative. In this study, sentiment analysis was applied to track the negative and positive sentiment tweets of users who also self-report as experiencing trauma or distress.

A word cloud is a visual representation of the frequency of words within a text corpus. In a word cloud, words are highlighted in accordance with their frequency. As part of this study, a word cloud containing frequently occurring words was generated using Python’s *wordcloud* library [40] and used to gain a deeper understanding of the tweets posted by Twitter users about PTSD during the COVID-19 pandemic. In particular, a word cloud can help to better understand the potential reason behind the peak of tweets about PTSD in January 2021 and the disproportionality toward the negative sentiment.

#### Association Rule Mining

Next, we used association rule mining, an unsupervised machine learning approach, to find dependence among terms and sentiment in the data sets [41]. This analysis aimed to examine the topics people were discussing during the pandemic when they talked negatively or positively about PTSD. In the association rule mining application, we treated all unigram and bigram terms as items and tweets as transactions. Our objective was to discover rules in the form X ⇒ Y, where X and Y are either 1-itemsets with 1 word, such as *mask* or 2-itemsets with 2 words, such as *wear mask.* We added new terms such as *positive_sentiment* and *negative_sentiment* to each tweet when the sentiment confidence score was >0.3 or <−0.3, respectively. We calculated 3 pattern evaluation metrics for the rule of X ⇒ Y for each conceivable combination of the itemsets of X and Y in the entire data set: support, confidence, and lift.

In association rule mining, it is crucial to set appropriate thresholds for these performance metrics to identify significant and intriguing rules. These thresholds can be determined by considering factors such as domain knowledge, measure distribution, data set size, and the desired level of confidence. However, the method for determining these thresholds is still largely based on user intuition [42]. In this study, we experimented by starting with conservatively high threshold values and then adjusting these 3 threshold values to obtain the top 5 strongest rules.

### Statistical Tests

The Kruskal-Wallis test is a nonparametric test in which normal distribution, interval data, and homogeneity of group variance are not among the assumptions to fulfill. As an alternative to a parametric 1-way ANOVA, this technique is flexible, convenient, easy to use, and powerful, and it can be used to measure differences among >2 groups. A Mann-Whitney *U* test, also known as a Wilcoxon rank sum test, can measure differences between 2 groups when the data are not normally distributed [43].

In this study, a Kruskal-Wallis test was first performed to analyze whether there was a significant difference in the retweet count and like count among all social support types. Next, to measure the difference in the retweet count and like count between paired social support types, we conducted post hoc Mann-Whitney *U* test.

### Ethics Approval

The institutional review boards of the University of North Texas (IRB-21-623) and Yale University (2000032441) reviewed and approved the study protocol, determining that it meets the criteria for exemption for research involving human participants.

### Privacy Protection Arrangements

Although discussing PTSD-related issues on social media could be distressing for some Twitter users, this study provides crucial insights into using social media to support individuals with PTSD during a pandemic, and our research followed strict research protocols. To protect the rights and privacy of Twitter users, we followed strict ethical principles throughout the study. All personally identifiable information that could reveal users’ identities was anonymized. Data collection, management, and analysis (except for content analysis) were conducted on a secure research cluster computer. Only a limited number (2225) of tweets were transferred to research team members’ password-protected computers and then transferred back to the secure server after analysis for the content analysis. No data were made available as open-access data. We were cautious when selecting tweets for publication to ensure that they did not put the tweet authors at risk; for instance, we used only partial tweets with unnecessary portions replaced by pseudorandom text for presentation purposes.

## Results

### Content Analysis

#### Overview

In this study, we examined the content of the tweets based on the 4 categories of social support developed by House [26]: *emotional*, *informational*, *appraisal*, and *instrumental*. A total of 2225 tweets were coded based on the social support types and an additional theme (*general comments and questions*). More than half (1214/2225, 54.56%) of the tweets contained content related to social support, whereas 45.44% (1011/2225) of the tweets contained only *general comments and questions*. According to the content analysis results, most of the tweets (947/2225, 42.56%) analyzed in this study indicated that people had been seeking and providing *emotional support*. *Informational support* was identified as the second most sought and provided social support type (128/2225, 5.75%), followed by *instrumental support* (117/2225, 5.26%). *Appraisal support* was the least commonly mentioned social support type (70/2225, 3.15%; Table 1).

**Figure 1:**
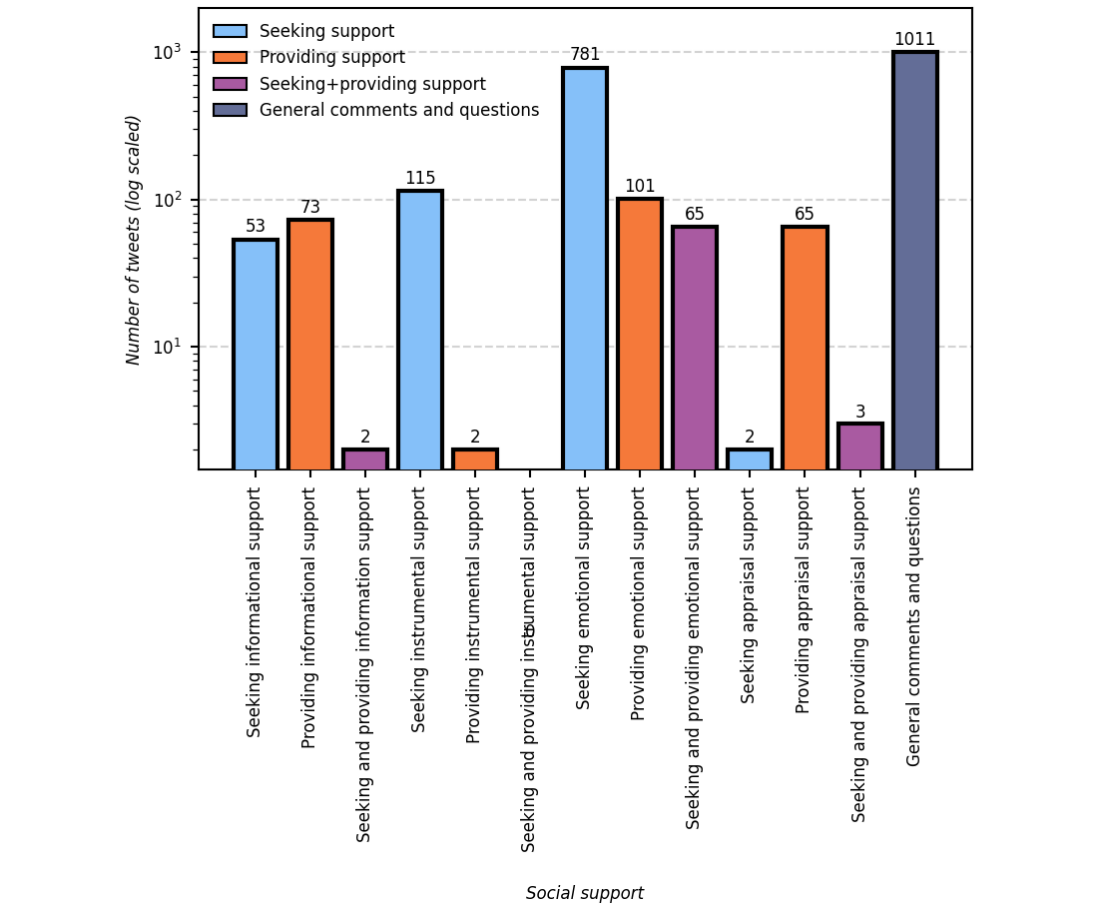
Number of tweets with self-reported trauma or distress across social support types during the COVID-19 pandemic.

Furthermore, 42.74% (951/2225) of the users *sought* social support, whereas 10.83% (241/2225) *provided* support for others on Twitter, meaning that people tended to seek social support more often than they provided it (Figure 1).

**Table 1:** Definition of social support types [26] and sample tweets (n=2225).

Category of social support	Description	Sample tweets	Values, n (%)
Informational support	Messages provide relevant advice or helpful knowledge to aid in coping with significant challenges faced within a particular context	“...I have ptsd and absolutely cannot wear a mask I am looking for info” “[A]nd option for those with ptsd officially diagnosed in 2004 involving masks because I still have to self-harm each time.”	128 (5.75)
Instrumental support	Messages include direct tangible help, such as assisting with a task or providing financial assistance	“I have been out of work due to my job shutting down during this pandemic I suffer from ptsd” “[T]hey don’t care about exemptions I’ve had people tell me to my face as they push me out the door I don’t have a job”	117 (5.26)
Emotional support	Messages include verbal or nonverbal displays of understanding, caring, kindness, trust, or love to a person in distress	“I think I have agoraphobia now combined with my ptsd I don’t even know how I’m going to work it out in...it’s just dark and gloomy you are not alone and there is nothing wrong with you this pandemic has been hard on us love” “I haven’t seen my family since last Christmas I’ve been waiting this entire pandemic to get emdr [eye movement desensitization and reprocessing] for my ptsd”	947 (42.56)
Appraisal support	Messages are distinct from informational support because the knowledge conveyed also helps recipients better evaluate themselves or their situations through social comparison	“I have been very angry all day. Yes, we need to heal. Between 45 and covid, we all have PTSD^a^ and need to deal with it. I have been using an app to meditate”	70 (3.15)

^a^PTSD: posttraumatic stress disorder.

#### Informational Support

##### Overview

The tweets (128/2225, 5.75%) in this category were posted by people who sought and provided relevant advice or helpful knowledge to aid in coping with trauma or distress during the COVID-19 pandemic. Of the 128 users, 53 (41.4%) sought *informational support*, whereas 73 (57%) provided *informational support*. Furthermore, 1.6% (2/128) both sought and provided *informational support*. People in this category were seeking and providing information about wearing masks, mental health status, and treatment; for example, as seen in the sample of tweets mentioning PTSD in the following subsection, people were struggling with wearing masks because this increased their anxiety levels as a result of trauma or distress.

##### Masks

Wearing a mask can lead to increased distress for some individuals. According to our analysis, some Twitter users were provided with information about the difficulty of wearing masks. People were experiencing headaches, dizziness, nausea, anxiety, and inability to breathe because of wearing masks during the pandemic. Thus, users whose tweets indicated trauma or distress were seeking information about how to manage the requirements of wearing masks in public places:

[T]his is what I’ve been having trouble understanding though I know this is true also mandate says that people with health problems don’t have to wear a mask I have medical documents for my ptsd severe anxiety wear it only in grocery what’s diff.

Sample tweet 1

I have ptsd and absolutely cannot wear a mask I am looking for info.

Sample tweet 2

I get sick with a mask on makes me dizzy nauseous bad headaches not to mention I have ptsd with claustrophobia.

Sample tweet 3

People also provided information on how to make wearing masks more comfortable for those experiencing trauma or distress:

[The mask] I own made it a tiny bit harder to breathe so what did I do find a different one that does not do that not being able to breathe is a trigger the mask was a neck gaiter and the one in my pic works better.

Sample tweet 4

Some people also claimed that they experienced trauma or distress during the pandemic because they had to wear masks and because they had also contracted COVID-19:

I have PTSD from wearing a mask in the hospital while my daughter had Covid. I caught it from her. I was super careful with mask wearing and hand washing. It made me wonder if masks were even helpful at all...Drs says we are all getting it eventually.

Sample tweet 5

Why is the only metric of severity calculated by death I had covid...now have ptsd from near death experience asthma and chronic headaches and I consider myself lucky people have severe heart and lung damage amputations strokes etc.

Sample tweet 6

##### Treatment

People indicating trauma or distress in tweets were providing some information regarding treatment methods to support their mental health during the pandemic. In addition, some therapy methods such as online therapy and self-guided therapy helped those experiencing trauma or distress, especially during drastic changes in their life:

@...I have ptsd...have you tried tele visits thanks to covid all my therapy is online now it’s great phone works for video calls too in most cases just have to find someone who does that.

Sample tweet 7

@...I’m trying to tackle my ptsd using workbooks and self-guided therapy. It’s a slow process...I’m learning will absolutely persist past the pandemic.

Sample tweet 8

Some methods to alleviate trauma or distress, such as listening to music, exercising, and reading books, were also shared:

@...just for myself simple exercise like walking getting good sleep & eating healthy all those things help the body stay balanced if that isn’t enough get professional help, I have ptsd but good help saved my life I’m a happy person managing even in the pandemic good luck.

Sample tweet 9

@...thank you @...you have saved my life twice now and i don’t know how to ever repay u for that I suffer with severe ptsd and depression and through these years your music has brought me light and hope being alone in lock down i have struggled to the point of giving up i love u.

Sample tweet 10

@...I was diagnosed with ptsd a week after the gyms reopened going to the gym has helped me so much it gives me focus now, I’m scared of not having that routine and where my thoughts will go gyms are more than just gyms for some people #mental health #gyms #lockdown #help.

Sample tweet 11

#### Instrumental Support

##### Overview

In addition to seeking or providing informational support, people self-reporting trauma or distress in tweets also asked for direct tangible help, such as assisting with a task or providing financial assistance. In other words, in this category, the tweets (117/2225, 5.26%) mostly expressed the financial challenges experienced by people during the pandemic. Most of the users (115/117, 98.3%) were seeking *instrumental support,* whereas 1.7% (2/117) provided *instrumental support*. Users talked about their financial loss because of having lost their job during the pandemic, which was why they were seeking *instrumental support*.

##### Job Loss

During the pandemic, several people lost their jobs. However, people struggling with trauma or distress especially had difficulties finding jobs because they could not adapt to the changes in the job environment and conditions because of COVID–19-related measures. Thus, they were seeking instrumental support on Twitter:

@...hello my name is...I am a military veteran...I was officially diagnosed with complex ptsd and cannot do a normal job I am self-employed and have lost all income to covid19 how am i being helped.

Sample tweet 12

@...I have been out of work due to my job shutting down during this pandemic I suffer from ptsd anxiety I am the last person who likes asking for help but if anyone wants to help me get some meds I would be beyond grateful for the kindness #plantbased.

Sample tweet 13

##### Financial Difficulties in Paying Rent and Bills

Twitter users included in this study were also seeking support and financial assistance to pay their bills and rent:

@...I’m jobless covid and have to take over a lease this month and was recently properly diagnosed with ptsd anything would help so much therapy has been expensive but so necessary bless anyone who feels kind enough.

Sample tweet 14

healthcaregov today you canceled my health care without my consent during a pandemic I won’t have coverage till dec 1st, and I have ptsd & anxiety disorder I see therapist 2x a week amp psychiatrist and you’re telling me I’m not covered you are monsters.

Sample tweet 15

#### Emotional Support

##### Overview

Among the Twitter users (947/2225, 42.56%) who reported *emotional support*, 82.5% (781/947) were seeking *emotional support*, whereas 10.7% (101/947) were providing *emotional support*, with 6.9% (65/947) both seeking and providing *emotional support*. These numbers show a huge disparity between the demand and supply of *emotional support*. Twitter users described it as pushing others away, longing for connection, loneliness, isolation, missing their families and friends, and losing family members.

##### Family

Twitter users who provided or sought *emotional support* did so because they had experienced trauma or distress during the lockdown, often leading to feelings of longing for their family members:

@...I haven’t seen my family since last Christmas I’ve been waiting this entire pandemic to get emdr [eye movement desensitization and reprocessing] for my ptsd you all don’t even pretend to care about us.

Sample tweet 16

##### Suicidal Behavior

A new term, *COVID PTSD*, was raised among Twitter users. Some of the people self-reporting COVID-19 and trauma or distress mentioned suicide attempts, and some showed suicidal behaviors because they could not handle the symptoms of trauma or distress as well as the pandemic-related stressors. Thus, these people shared their situation and sought *emotional support* on Twitter to overcome suicidal behavior:

@...I tried to kill myself a few months ago failed...I’m under a crisis team and the community mental health team I have ptsd from a previous trauma and fairly recently diagnosed with ms treatment ceased with covid19.

Sample tweet 17

@...I read an article that children are being diagnosed with what the shrinks are calling covid ptsd and some as young as 10 have committed suicide its heartbreaking it really is.

Sample tweet 18

##### Masks

Some Twitter users included in this study also sought emotional support to avoid wearing masks in public places because masks exacerbated their mental health symptoms:

@...I have ptsd if I wear a mask, I will become seriously ill I try to stay at home as much as I can, but I do occasionally need to go out I am terribly sorry if that inconveniences you, but I also deserve to go to the park.

Sample tweet 19

##### Health Care Workers

There were also health care workers facing trauma or distress owing to the pandemic. Most of the health care workers had witnessed patients dying from COVID-19 infection. Therefore, they were supporting each other on Twitter by sharing that they faced the same situations in the hospitals:

@...I have suffered from covid since April had covid plus I’m a healthcare worker I have lung damage anxiety depression and ptsd plus I missed so much work I had to deplete my savings.

Sample tweet 20

@...I just get off work I am respiratory therapist at a level one hospital I work at...medical center in...I have ptsd from the covid from what I saw you bring me joy you give me hope I almost lost hope thank you.

Sample tweet 21

@...I have ptsd from being a respiratory therapist in the heart of the covid pandemic I saw so many people die horribly plus I ended up with covid...I have horrible nightmares of my patients’ dying faces hang in there you are not alone.

Sample tweet 22

##### Isolation

Some users also felt isolated and provided support to each other to deal with the loneliness:

@...we are on lock down now in this part of...I will if we ever get back to normal sometimes, I wake up and feel it’s a dream, I have ptsd so this year not been a good one for me.

Sample tweet 23

[same] @...I think I have agoraphobia now combined with my ptsd I don’t even know how I’m going to work it out in...it’s just dark and gloomy you are not alone and there is nothing wrong with you this pandemic has been hard on us love.

Sample tweet 24

#### Appraisal Support

Twitter users (70/2225, 3.15%) also expressed the need for *appraisal support*. This category concerned how people helped each other, regarding trauma or distress, to evaluate their situation with regard to coping with the COVID-19 pandemic. Of the 70 users, 65 (93%) provided *appraisal support,* whereas only 2 (3%) sought *appraisal support*. Users also described their feelings and current situations both to evaluate themselves in terms of coping with PTSD and the pandemic and to provide support to others experiencing trauma or distress:

@...im sure I’m so sorry you’re going through all of that I have ptsd myself and its made the pandemic more scary writing has been a huge help so I’m glad we both have writing and friends to find strength in that’s awesome about the book I’m looking forward to it.

Sample tweet 25

@...I have ptsd and am triggered by my face and neck being touched having something on my face can be very overwhelming there’s times I’m in stores sweating bc [because] I’m struggling so much I am not emotionally equipped to deal with this but i still wear a mask.

Sample tweet 26

### Sentiment Analysis

#### Overview

**Figure 2:**
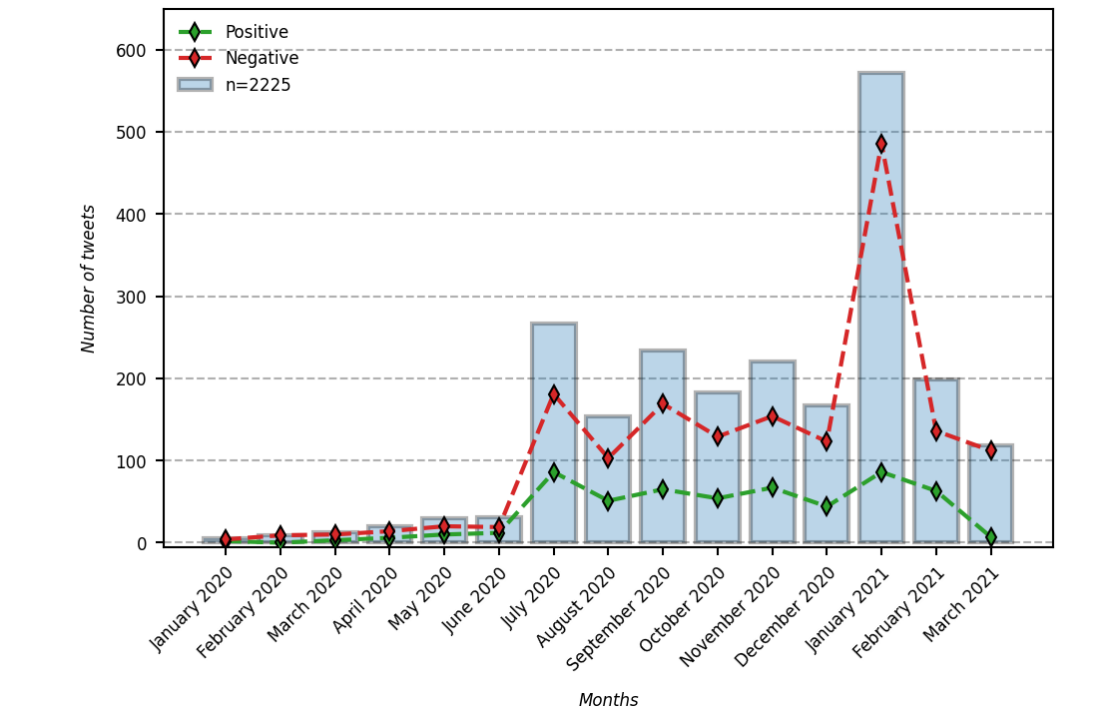
Monthly distribution of positive and negative sentiment tweets.

The positive and negative sentiment tweets posted between January 1, 2020, and March 15, 2021, were analyzed. The analysis showed that, during the COVID-19 pandemic, the people included in this study tended to post mostly negative sentiment tweets (1669/2225, 75.01%), whereas fewer tweets (556/2225, 24.99%) presented positive sentiment. Figure 2 presents the changes of sentiment in selected tweets over the course of the pandemic. Tweets with negative sentiment outnumbered those with positive sentiment in all months. The findings also showed that people whose tweets mentioned trauma or distress used Twitter in January 2021 (572/2225, 25.71%) more actively than in other months. In January 2021, the proportion of negative sentiment tweets was also high.

#### Word Cloud

A word cloud was created to visualize the frequently used words on Twitter between January 1, 2021, and January 31, 2021. Of the 2225 tweets, 572 (25.71%) were posted in January 2021, with 15% (n=86) expressing positive sentiment and 85% (n=486) expressing negative sentiment. Some of the most frequent words were: *ptsd, mask, wear, express, living, existing, need, human, connections, oxygen, and thief.*

Some terms such as *human connections and need human* showed that people struggling with trauma or distress may have been experiencing psychological or emotional troubles as a consequence of lockdowns and physical distancing. In addition, *wearing mask, oxygen thief, and oxygen release* were the other terms used most often in Twitter posts during this period. Therefore, these terms may be reflections of the challenges involved in wearing masks in daily life (the word cloud can be found in Multimedia Appendix 1, and the top 30 most frequent words from the word cloud are presented in Table 2).

**Table 2:** Top 30 most frequent words from the word cloud with their frequency.

Rank	Words	Frequency (N=5224), n (%)
1	ptsd	534 (10.22)
2	wear mask	247 (4.73)
3	express	207 (3.96)
4	living existing	206 (3.94
5	need human	206 (3.94)
6	human connections	206 (3.94)
7	told wear	206 (3.94)
8	wearing mask	95 (1.82)
9	legitimate reason	91 (1.74)
10	oxygen thief	91 (1.74)
11	oxygen release	91 (1.74)
12	mask autistic	91 (1.74)
13	asthmatic	91 (1.74)
14	diagnosed	90 (1.72)
15	covid	79 (1.51)
16	https	47 (0.90)
17	low	47 (0.90)
18	pandemic pandemic	44 (0.84)
19	test scores	44 (0.84)
20	pandemic black	44 (0.84)
21	kids inner	44 (0.84)
22	Black kids	44 (0.84)
23	inner city	44 (0.84)
24	ptsd pandemic	44 (0.84)
25	pandemic test	42 (0.80)
26	anxiety	41 (0.78)
27	amp	37 (0.71)
28	now	33 (0.63)
29	march	28 (0.54)
30	year	27 (0.52)

#### Association Between Topic and Sentiment (Association Rule Mining)

We applied the association rule mining technique to discover strong relationships between terms that could represent the topics of interest related to PTSD and their corresponding sentiment. We used 3 interestingness measures as thresholds (lift >3.5, support >0.1, and confidence >0.4) to present strong associations. Table 3 shows the association strengths in descending order of lift. Only rules, including sentiment, are presented in Table 3 because the goal is to find associations between topic and sentiment. The strongest association was {Get, Mask ⇒ Negative, Wear: 4.39}, followed by {Negative, Wear ⇒ Get, Mask: 4.39} and {Wear ⇒ Negative, Get, Mask: 4.31}. Setting a threshold that is too low results in a large number of noisy rules. Conversely, if the threshold is set too high, a significant loss of information can occur. Therefore, although there may be important rules not accounted for by the current threshold values, Table 3 presents the strongest signals. Among the rules passing the thresholds, all terms were mask- and negative sentiment–related. This finding suggests that people were experiencing extreme stress regarding wearing masks and supports the findings from the content analysis.

**Table 3:** Suggested association rules among terms and sentiment.

{X}	{Y}	Lift	Support	Confidence
{Get, Mask}	{Negative, Wear}	4.39	0.11	0.95
{Negative, Wear}	{Get, Mask}	4.39	0.11	0.49
{Wear}	{Negative, Get, Mask}	4.01	0.11	0.43
{Negative, Get, Mask}	{Wear}	4.01	0.11	0.99
{Get, Wear}	{Negative, Mask}	3.65	0.11	0.96

### Kruskal-Wallis and Mann-Whitney U Tests: Analyzing the Retweet Count and Like Count

The retweet count and like count were also analyzed to explore people’s endorsements of different types of social support. Thus, the Kruskal-Wallis tests were applied first to see whether there was a difference in people’s endorsements (ie, the retweet count and like count/tweet) depending on the different social support types. The results indicated that the means were significantly different across the groups; at least 1 of them had a different median than the others (*P*<.001). *Emotional support* showed the largest number of retweets and likes, whereas *instrumental support* showed the least number of retweets and likes. This is an indication that people did not display the same reactions to tweets related to each of the 4 social support types by retweeting them or liking them.

Finally, the Mann-Whitney *U* test was conducted to determine whether there was a difference in the retweet count and like count between all pairs of different social support types. The results indicated a significant difference (Table 4) between *emotional support* and other social support types (all *P*<.05), besides *informational support* (*P*>.05). There was a more significant difference in retweet counts than in like counts. A like count is a passive form of endorsement that only requires clicking or tapping a button. By contrast, the retweet count is a more active form of endorsement because the user acknowledges the retweeted content and often shares it with their followers. In conclusion, during the pandemic, people engaged more often in *emotional support* than *appraisal support* or *instrumental support*.

**Table 4:** Comparing the retweet count and like count of tweets based on social support type.

Compared social support types			Retweet count		Like count
**Informational support vs appraisal support**					
	Informational support, mean (SD)	1.2 (2.6)		2.2 (4.4)	
	Appraisal support, mean (SD)	0.3 (1.2)		2.8 (6.1)	
	Mann-Whitney *U* test	3695.5		4164.0	
	*P* value	.001		.17	
**Informational support vs instrumental support**					
	Informational support, mean (SD)	1.2 (2.6)		2.2 (4.4)	
	Instrumental support, mean (SD)	0.1 (0.7)		2.3 (7.2)	
	Mann-Whitney *U* test	6082.5		6872.0	
	*P* value	<.001		.10	
**Emotional support vs informational support**					
	Emotional support, mean (SD)	4.3 (22.8)		13.4 (290.9)	
	Informational support, mean (SD)	1.2 (2.6)		2.2 (4.4)	
	Mann-Whitney *U* test	57,012.0		57,853.0	
	*P* value	.051		.15	
**Emotional support vs instrumental support**					
	Emotional support, mean (SD)	4.3 (22.8)		13.4 (290.9)	
	Instrumental support, mean (SD)	0.1 (0.7)		2.3 (7.2)	
	Mann-Whitney *U* test	48,948.0		47,930.0	
	*P* value	<.001		.002	
**Emotional support vs appraisal support**					
	Emotional support, mean (SD)	4.3 (22.8)		13.4 (290.9)	
	Appraisal support, mean (SD)	0.3 (1.2)		2.8 (6.1)	
	Mann-Whitney *U* test	29,652.0		29,226.0	
	*P* value	.01		.02	
**Appraisal support vs instrumental support**					
	Appraisal support, mean (SD)	0.3 (1.2)		2.8 (6.1)	
	Instrumental support, mean (SD)	0.1 (0.7)		2.3 (7.2)	
	Mann-Whitney *U* test	4047.0		4089.5	
	*P* value	.38		.49	

## Discussion

### Principal Findings

The overall impact of the COVID-19 pandemic on the mental health of the world’s population has yet to be realized. The prevalence of mental health conditions, such as PTSD, has significantly increased. During the pandemic, physical distancing requirements led to an increase in the use of social media as a way to connect and to seek and provide social support. The results from the content analysis showed that most of the tweets mentioning PTSD (947/2225, 42.56%) indicated that individuals were seeking or providing *emotional support*, followed by tweets expressing *informational support* (128/2225, 5.75%), *instrumental support* (117/2225, 5.26%), and *appraisal support* (70/2225, 3.15%). Furthermore, tweets that mentioned PTSD and *emotional support* received more endorsements than tweets expressing other types of social support. Most of the tweets (1699/2225, 76.36%) were found to be negative, and association rule mining showed the particularly negative attitude of people toward wearing a mask.

In regard to *emotional support*, the reduction of in-person social interactions during the pandemic led to users seeking *emotional support* on social media platforms (eg, Twitter). Loneliness is a serious global issue that the pandemic exacerbated. During the pandemic, people were grieving the loss of loved ones and their loss of normalcy, as well as lamenting canceled plans. Social media can be a good channel for providing *emotional support* during lockdowns. The Kruskal-Wallis tests were performed to determine whether there was a significant difference in users’ endorsements among all social support types by comparing the retweet count and like count of tweets. The results showed the importance of *emotional support* because this type of support was accompanied by the most endorsements by Twitter users experiencing trauma or distress. However, the disparity between *providing emotional support* and *seeking emotional support* showed a serious lack of social support for this group. One insight gained from this study was that the *retweet* count, which is indicative of a more active form of endorsement, might better reflect users’ endorsement than the *like* count. Choi et al [44], who conducted the content and statistical analyses of YouTube videos, found that commenting reflects a higher level of engagement than viewing or liking because, for commenting, people need to feel the urge to express their opinions and feelings publicly. Although retweeting is different from commenting, they are similar in that both involve stronger engagement than simply liking a tweet.

As the Twitter users included in this study sought informational support about mask wearing and mental health care, an important finding was that wearing masks can intensify PTSD symptoms for those who have experienced trauma related to their mouth or face being covered. This finding is supported by both the association rule mining and sentiment analyses over time. Twitter users experiencing trauma or distress had concerns about mask wearing. Although the mask mandate was necessary to curb the spread of SARS-CoV-2, our recommendation is that the policy needed to be fine-tuned for individuals who have experienced trauma that prevents them from comfortably wearing a mask.

The results of the sentiment analysis revealed that, in general, there were more negative sentiment tweets than positive sentiment tweets. In particular, there was a vast difference between negative sentiment tweets and positive sentiment tweets in January 2021. We observed a large spike in negative sentiment tweets in January 2021. In the United States, President Joe Biden signed an executive order on January 20, 2021, requiring “masking and physical distancing in federal buildings, on federal lands, and by government contractors [45].” This was followed by the president signing 10 executive orders on January 21, 2021, to curb the spread of SARS-CoV-2, including one that required Americans to wear masks at airports and in airplanes, ships, city buses, trains, and other forms of public transportation [46]. In addition, the Centers for Disease Control and Prevention issued an order on January 30, 2021, requiring travelers on airplanes and public transportation (eg, buses and subways) to wear masks [47]. A word cloud of tweets from January 2021 revealed negative sentiments such as *oxygen thief* and *mask autistic*. Significant discussions among individuals about the challenging part of wearing masks in daily life were also discovered in the content analysis.

When providing resources to people seeking emotional support (eg, access to mental health services and resources as well as online support groups), we must also consider how the policies that are being implemented will affect the lives of those experiencing trauma or distress. During the pandemic, emotional support and informational support surfaced as the top forms of support needed—the need to know that someone cared and knowledge of how to cope (eg, seeking information about wearing masks and mental health resources). There was a lot of controversy about physical distancing and mask mandates during the pandemic. Our findings indicate that wearing a mask can act as a trigger for individuals struggling with trauma and that health care workers also experienced extreme trauma or distress while working on the front line in the battle against COVID-19. Future public health campaigns should include strategies to address these needs.

### Limitations

This study has some limitations. First, some tweets related to symptoms of PTSD (as a proxy measure of trauma or distress) and social support during the COVID-19 pandemic may have been missed in the study. Although we used carefully selected terms to extract data related to COVID-19, this study might have missed some relevant discussions on Twitter using different terms than the ones we chose. Another limitation includes its focus solely on Twitter data, which may not be representative of all individuals’ views and experiences regarding trauma or distress. Twitter users are a subset of the population, and those who do not use social media and Twitter may have different experiences and needs. Furthermore, the study could not report on the demographics of the Twitter users, such as age, gender, and ethnicity, which could affect the findings and limit their applications.

The tweets selected in this study mentioned self-reports of trauma or distress; however, we were not able to verify the mental health history of the Twitter users. Therefore, it is critical to consider these limitations when interpreting the results. Given the prevalence of social media use, understanding the risks associated with seeking support on the web is critical for individuals experiencing trauma, distress, and other mental health conditions. In addition, further research is needed to gain a more comprehensive understanding of the challenges and needs of individuals living with trauma, distress, and PTSD in various contexts. This will help to identify common challenges and inform the development of more effective interventions and support services for individuals who experience mental health challenges during a pandemic.

### Conclusions

The COVID-19 pandemic had a significant impact on individuals dealing with trauma or distress because it led to increased levels of stress, uncertainty, and fear. The restrictions during the pandemic also led to social isolation and increased feelings of loneliness, which can be especially difficult for individuals experiencing mental health stressors leading to trauma or distress. In addition to these challenges, as our findings showed, people were also struggling with wearing masks during the pandemic because mask wearing can cause trauma or distress for some individuals. Furthermore, because the increased stress and isolation as well as the requirement of wearing masks exacerbated trauma or distress and made it more difficult for individuals to physically access social support, our work showed the potential of social media platforms such as Twitter to be used as a source of social support. Our study also indicated that, during the pandemic, people generally sought and provided emotional support on Twitter regarding the challenging aspects of wearing masks, mental health status, financial difficulties, and treatment methods for mental health conditions. This study makes an important contribution to understanding the special needs of individuals who experienced trauma or distress during the pandemic.

## Appendix

Multimedia Appendix 1 Word cloud of frequently used words on Twitter in January 2021.

## Abbreviations

API: application programming interface
IMDb: Internet Movie Database
PTSD: posttraumatic stress disorder
SARS: severe acute respiratory syndrome

## Data Availability

The data sets collected and analyzed during this study cannot be made available owing to ethical considerations. Sharing the data may raise the possibility of revealing the identities of the Twitter users included in this study, which could put individuals at risk for harassment and discrimination.
