# Supplementary figures and images for: Seeking and Providing Social Support on Twitter for Trauma and Distress During the COVID-19 Pandemic: Content and Sentiment Analysis
Source: J Med Internet Res. 2023 Aug 31;25:e46343. doi: 10.2196/46343 (PMC10502591; doi:10.2196/46343)

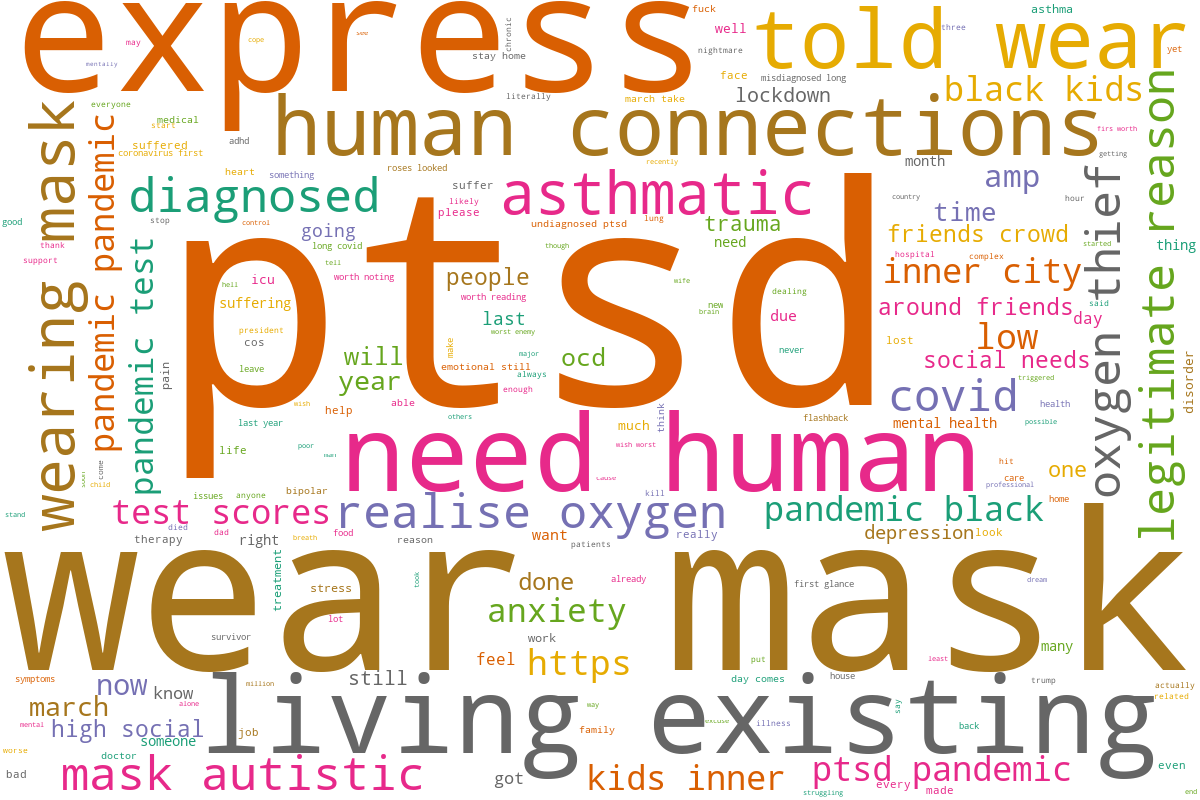

Supplement: Multimedia Appendix 1 [file jmir_v25i1e46343_app1.png]
